# The Effects of Gender Signals and Performance in Online Product Reviews

**DOI:** 10.3389/fdata.2021.771404

**Published:** 2022-01-07

**Authors:** Sandipan Sikdar, Rachneet Sachdeva, Johannes Wachs, Florian Lemmerich, Markus Strohmaier

**Affiliations:** ^1^ RWTH Aachen University, Aachen, Germany; ^2^ Vienna University of Economics and Business, Vienna, Austria; ^3^ Complexity Science Hub, Vienna, Austria; ^4^ University of Passau, Passau, Germany; ^5^ GESIS - Leibniz Institute for the Social Sciences, Cologne, Germany

**Keywords:** gender disclosure, online product reviews, matching, natural experiment, classification

## Abstract

This work quantifies the effects of signaling gender through gender specific user names, on the success of reviews written on the popular amazon.com shopping platform. Highly rated reviews play an important role in e-commerce since they are prominently displayed next to products. Differences in reviews, perceived—consciously or unconsciously—with respect to gender signals, can lead to crucial biases in determining what content and perspectives are represented among top reviews. To investigate this, we extract signals of author gender from user names to select reviews where the author’s likely gender can be inferred. Using reviews authored by these gender-signaling authors, we train a deep learning classifier to quantify the gendered writing style (i.e., *gendered performance*) of reviews written by authors who do not send clear gender signals *via* their user name. We contrast the effects of gender signaling and performance on the review helpfulness ratings using matching experiments. This is aimed at understanding if an advantage is to be gained by (not) signaling one’s gender when posting reviews. While we find no general trend that gendered signals or performances influence overall review success, we find strong context-specific effects. For example, reviews in product categories such as Electronics or Computers are perceived as less helpful when authors signal that they are likely woman, but are received as more helpful in categories such as Beauty or Clothing. In addition to these interesting findings, we believe this general chain of tools could be deployed across various social media platforms.

## 1 Introduction

Reviews play an important role in influencing purchasing decisions in online platforms (Von Helversen et al., 2018; Lee et al., 2019; Rajani and Nakhat, 2019). However, with several hundreds of reviews being written on some products on popular e-commerce websites like amazon.com, users often make purchasing decisions based on the *top-k* reviews. Helpfulness scores (e.g., upvotes and downvotes) play a key role in ranking these reviews (Yang et al., 2015; Tsaparas et al., 2011; Ghose and Ipeirotis, 2010). In this work, we analyze how users of the popular online shopping platform amazon.com rate the helpfulness of online product reviews depending on gendered signals and performance of their authors. Given that these ratings impact the visibility of reviews and hence their influence on product success, gender bias may shape the market in unexpected ways (boyd and Crawford, 2012). Differences in social outcomes between genders have been studied in many diverse online contexts including Wikipedia (Wagner et al., 2015), social media (Nilizadeh et al., 2016), online newspapers (Jia et al., 2016), and online freelance marketplaces (Hannák et al., 2017). Despite this previous work, it is still unclear how differences in outcomes relate to responses to signals of gender identity or to the gendered behavior implicitly revealed in content.

Existing literature has also mostly focused on understanding how genders are perceived across different online platforms Wagner et al. (2015); Hannák et al. (2017); Wachs et al. (2017); Jia et al. (2016). Often, it is assumed that the genders are declared for each individual on the platform. However, in practice users often tend to hide their gender—consciously or unconsciously—by using pseudonyms as their visible user names. Hence in contrast to the existing literature, we investigate how a contribution of an individual is perceived when the author has signaled his/her gender through user name vis-a-vis when the gender is not clear but only performed (i.e., gendered behavior observed through review text). In particular, we intend to check whether there is an advantage to be gained by not explicitly signaling one’s gender when creating content in an online platform *via* the user name. To the best of our knowledge, this aspect has received little attention in literature so far.

In this paper, we study the effect of gender on the helpfulness scores of amazon review data. We differentiate between the effects of explicit gender indicators (*gender signals*) and the effects of gendered behavior (*gender performance*). For that purpose, we compare content by users signaling a likely gender in their user name to the case when no reliable inference about an author’s gender can be made that way. Additionally, we infer the gendered performance of all reviewers, including those who do not signal their likely gender *via* their user names, using data extracted from the text of the reviews. Thus, our analysis focuses on two specific research questions. First, is there a difference in the success of product reviews, measured by ratings of “helpfulness” made by other users, depending on the signaled gender of the author? Second, is there an effect on the appreciation of reviews if the gender of the author is not explicitly signaled, but performed?

**FIGURE 1:**
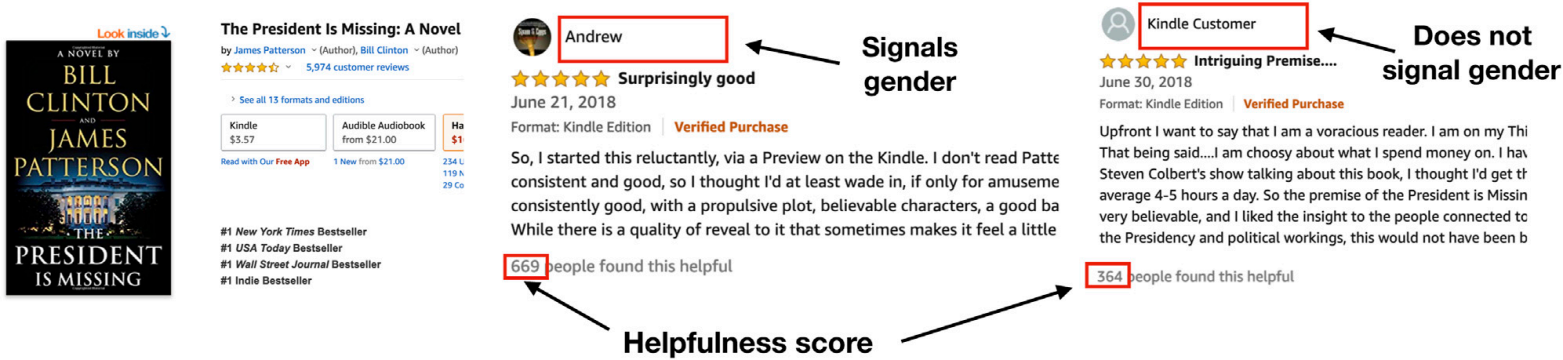
Illustration: Consider the reviews written for the product “The President is Missing: A Novel.” The author of the review on the left signals a likely gender by the user name “Andrew.” On the right the author’s user name “Kindle Customer” does not signal a likely gender. Further, the perceived helpfulness of a review can be quantified by the helpfulness score of the review. We explore whether signals of the gender of the authors influences the perceived helpfulness of a review.

To illustrate our setting, we show two example reviews in Figure 1. In the review on the left we observe a signal of the author’s likely gender *via* his user name (“Andrew”). In contrast, we cannot reasonably infer the likely gender of the author of the review on the right. Other users on Amazon can express appreciation for a review by marking it as helpful. The number of such appraisals of a review, called its “helpfulness score,” is displayed below each review. We investigate if there is a relationship between user gender signals and the helpfulness score the review receives. If there is a relationship between gender disclosure and feedback, how does it vary across the kinds of products reviewed on Amazon?

We study a dataset with more than 80 million reviews written by around 21 million users about 9 million products on “Amazon.com”^1^, Amazon’s North American domain. Using the name-to-gender prediction tool *Gender-Guesser* ^2^, we are able to label the gender of reviewers for 42% of the total reviews in the dataset that are in line with human judgment in 98*%* of cases.

With these signaled gender labels, we employ character-level Convolutional Neural Networks (CNN) as state-of-the-art text classification methods to measure the gendered behavior (performance) of reviews that do not signal gender *via* their user name. Note that the term gender performance is used to quantify the gendered behavior which we achieve through this gender classifier. We can infer the signaled gender of a user in a held-out test set with an overall accuracy 82% using the text features. We consider users who explicitly indicate their gender through chosen user names as *Signaling gender*. Similarly, users, whose gender cannot be reliably predicted from the user names, but can be predicted from the review text are considered as *performing gender*. Using this measure of performed gender, we categorize user reviews into four groups: *1*) signaling men, *2*) signaling women, *3*) performing men, *4*) performing women. We then perform a set of matching experiments to compare the helpfulness scores of reviews in the different groups in otherwise (e.g., with respect to publication time, review length, sentiment, product rating, etc.) similar reviews and study differences in social feedback these users receive across product categories.

We find that on average across all categories, gender signaling does not have an effect on the perceived helpfulness of product reviews. However, we observe substantial context-specific effects. For example, reviews authored by signaling women are perceived as more helpful in products related to categories like Movies or Beauty. By contrast, we notice increased helpfulness scores for signaling men in categories such as Electronics or Kindle. Comparing signaling women with performing women, we find that signaling gender hurts in categories such as Electronics, Games or Computers. Similar negative effects are observed for signaling vs performing men in product categories including Clothing, Beauty or Toys. We consider the presence of such context-sensitive effects as a primary finding of this paper that significantly extends previous findings in the field. Our results promote increased awareness of gender-specific effects in the perception of online reviews and suggest implications for online platforms (e.g., regarding the ranking of reviews or display of user names if they potentially imply gender) and their users (e.g., awareness of such effects when choosing a user name).

## 2 Dataset Preparation

This section describes the data we use in this study and introduces the methods we employ, to approach our research questions. In particular, we describe how the gender of reviewers can be inferred from user names and review content.

### 2.1 Dataset

We leverage a publicly available dataset of Amazon product reviews^3^ consisting of English language reviews written between May 1996 and July 2014 (McAuley et al., 2015; He and McAuley, 2016). Each review contains information on the author’s user name, the product rating (between one and five stars), a helpfulness score (i.e., the number of users who marked the review as helpful), the reviewed product, the date of the review, and its text. Each product is linked to meta-data including a description of the product, category information (each product can belong to multiple categories), price, brand, and image features.

The dataset contains about 80 million reviews (excluding around 2 million reviews with missing attributes) of 20.9 million unique reviewers about 9.01 million products assigned to 18.1 thousand categories. On average, each review contains 84.8 words and rates the product with 4.16 stars. Regarding helpfulness, reviews receive on average 2.07 upvotes and 0.71 downvotes. Information on the gender is not directly available from this dataset, and we will discuss the methods for inferring gender signals in the following section.

### 2.2 Gender Signals Though User Names

We now describe how we infer the perceived, signaled gender of reviewers. In general inferring the gender of online users is known to be a challenging task (Karimi et al., 2016; Lin and Serebrenik, 2016). To alleviate this problem, we first make the simplifying assumption of binary gender (see Section 5.2). We therefore identify users as likely (signaling) men or likely (signaling) women by applying the name-based gender prediction tool *gender-guesser* ^4^. Name-based methods have been effectively applied to measure demographics in a variety of online contexts (Mislove et al., 2011). Recent work using eye-tracking software suggests that individuals evaluating content online do look at names and photos of authors (Ford et al., 2019).

Gender-guesser is a dictionary of over 40,000 first names (collected from a variety of countries and regions) and their most likely binary gender, sourced from public statistics of names and sex recorded at birth. Specifically, each name contained in the dictionary is described as *male*, *female*, *mostly male* or *mostly female*. We apply gender-guesser to the first token in each user name. We ignore the inference for the latter two categories, classifying reviewers with “mostly male” and “mostly female” names as having unknown gender. To widen the scope of our inference, we also consider a manually collected list of keywords that give a clear indication of a specific gender, but are not given names (such as “*girl*” or “*woman*” for women and “*boy*” or “*dude*” for men).

Using this procedure, we classify from the 20.9 million unique reviewers approximately 5.43 million (26.0*%*) as likely signaling men, and 5.6 million (26.8*%*) as likely signaling women. These account for around 18.9 million and 19.3 million reviews respectively. The remaining reviewers (about 11.03 million or 47.2*%*, accounting for 41.1 million reviews) do not signal a likely gender with their user name. As we are interested in how the perceived gender of a reviewer relates to the social feedback given by other users, we believe that our approach, i.e., to infer gender from the displayed name gives a mostly accurate picture about how other users perceive the gender of a reviewer.

#### Comparison With Human Gender Perception of User Names

We confirm this assumption of user name categorization reflecting human gender perception in an experiment. For this purpose, we provided six human annotators with 500 user names that had been labeled with a gender by our procedure, and ask them to guess the genders. For a given user name the annotators were given three choices - *1*) “male,” *2*) “female” and *3*) “can’t say.” Responses of all six annotators were recorded for every user name. The annotators achieved an overall good inter-annotator agreement (“Fleiss”-kappa’ of 0.81) for this task. In case of disagreements between annotators, we assigned a label for human gender perception based on majority voting. Comparing these labels with the results of our automatic procedure, we observe a match of 98% (490 out of 500). Additionally, we asked human annotators to assign a gender to 500 random user names, which we could not automatically classify. In this case, human annotators are unable to detect gender from names in 90% of the cases (“Fleiss”-kappa’ of 0.8). Inspection of cases where human annotators were able to guess the gender from these names show mostly names with unorthodox spelling but similar phonetics (e.g., “Florentyna” instead of “Florentina”). This suggests a future line of research to improve name-based gender inference tools.

Together, the above results indicate that our automatic procedure is well in line with human perception for detecting genders from user names, i.e., if we can infer a gender based on the user name automatically, the gender signal contained in a user name is strong, while there is no clear indication of the gender from the user name if we cannot assign a gender label automatically. In the remainder of the paper, we therefore differentiate between two groups of reviewers (and by implication, reviews):

• Signaling Men and Women: This is the set of reviewers for which we can automatically infer the gender from the user name, i.e., the user name sends a clear signal about the likely gender of the reviewer.
• Non-signaling users: This is the set of reviews for which we cannot infer the gender from the user name. As shown by our experiments, humans do not pick up clear gender signals for these users either.

Note that we assign here a gender label based on the gender perception of the user name, i.e., we assume a review written under a name with a likely gender is a strong signal of gender identity. While there are exceptions, we believe this is a reasonable assumption that has been adopted previously in multiple studies (Mislove et al., 2011; Ciot et al., 2013; Liu and Ruths, 2013), *see* also Section 5. Another point of validity for our assumption is that we are concerned with social feedback of other users in response to the signals contained in the user names. For the remainder of this paper, when we mention the signaled gender of a review, we would essentially mean the signaled *likely* gender of the reviewer who authored the review unless specified otherwise.

### 2.3 Measuring Gender Performance

**FIGURE 2:**
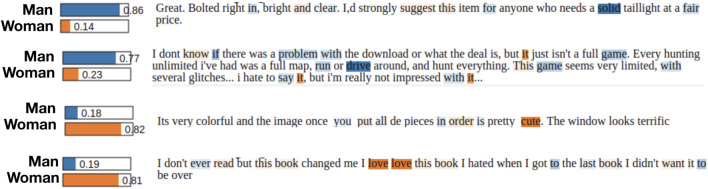
Distinctive writing patterns of inferred men and women. The first two examples are cases where the LogReg model predicts that the author is a signaling man with high probabilities (0.86 and 0.77 respectively) while the classifier predicts that the second pair of texts were very likely written by signaling women. Noticeably, the model gives more weight to words such as “solid,” “drive” etc. for the first two cases where the model predicts the gender. For the next two examples the model gives more weight to words such as “love,” “cute” etc. These exploratory results suggest that there exist differences in writing styles between gender signaling men and women while reviewing a product on Amazon.

In this work, we are interested in the effect of explicit gender signal on the perceived helpfulness. Since differences in helpfulness scores between signaling men and women can originate from these direct signals or from different underlying gendered behavior (*gender performance*), we next aim to model gender performance in review texts. For that purpose, the set of reviews with gender-signaling authors can be used as ground-truth to train machine learning models that infer the likely gender of the author from review texts. These would pick up on the gendered behavior embedded in the text of the review including style and word choice. However, designing such a classifier is only feasible if there is indeed a noticeable difference in the writing patterns of men and women. For illustration, we first train a simple logistic regression classifier (LogReg) on the ground truth (gender-signaling) set of reviews. Then, by utilizing the LIME framework (Ribeiro et al., 2016) we compare the reviews which are inferred by the classifier as (authored by) men with those inferred as women. In Figure 2 we present four such examples (two in each class). For the reviews predicted as written by signaling men, the classifier gives higher weights to words like “solid,” “drive” and “game,” while words such as “cute” and “love” are strong predictors that a review was written by a signaling woman. This goes to show that our collection of signaling men and women have distinctive writing styles which could be leveraged to design machine learning classifiers for inferring gendered behavior or performance from text. Note that several frameworks have been developed to interpret inference results (Lundberg and Lee, 2017; Shrikumar et al., 2017; Sundararajan et al., 2017) of machine learning models and could be deployed to the problem at hand. However, our motivation for the experiment was to investigate whether a classifier is able to identify linguistic differences between reviews written by a man and a woman when trained on the task of gender prediction from text and not to explain the inference results or obtain better explanations. Hence, we did not explore other frameworks.

In that direction, we now train a variety of machine learning models on the review text of reviews with disclosed gender (i.e., for which a gender is apparent from the user name) and apply the best performing model on the review text of reviews with undisclosed gender (i.e., for which the user name does not send a clear gender signal). The underlying idea is that we can derive the gendered behavior of users in many cases with high accuracy using machine learning techniques even if the underlying patterns used for classification cannot be picked up by humans.

#### Machine Learning for Inferring Gender From the Review Text

In general, we aim to train a model on the review text of reviews with disclosed gender (i.e., for which we could identify a gender label based on the user name) and apply this model to infer a gender label for undisclosed reviews. For this text classification task, we considered a variety of traditional machine learning models such as Logistic Regression (Fan et al., 2008) and Linear SVM (Fan et al., 2008), or XGBoost (Friedman, 2001) as well as state-of-the-art Deep Learning methods based on Recurrent Neural Networks (GRU (Cho et al., 2014)) or on Convolutional Neural Networks (CNNs) (Kim, 2014; Zhang et al., 2015). After extensive experiments, we focused on character-level CNNs since they offered the best predictive performance for our task by a small margin, and have also previously been shown to perform well for NLP tasks in general and classification tasks in particular (refer to Table 1 for the performance comparison of the models). We describe the detailed setup of this model next.

**TABLE 1:** Accuracy of different machine learning models on the task of inferring gender from text.

	Model	XGBoost	Log-Reg	RNN	CNN
Accuracy	without majority voting	0.708	0.691	0.733	0.752
	with majority voting	0.784	0.765	0.814	0.821

Character-level CNNs perform best among the models.

The vocabulary for this model is elementary and consists of only 69 characters which include 26 English alphabets, 0–9 digits and 33 other special characters. As input, the text is quantized with the help of this vocabulary. For each review, we consider a maximum text length of 1014 characters (which is sufficient to cover most of the reviews), dropping exceeding characters or padding missing ones, cf. (Zhang et al., 2015). We train the model with a batch size of 512. The model itself consists of overall nine layers, i.e., six convolutional layers and three fully connected layers. As suggested in the paper (Zhang et al., 2015), the first two and sixth convolutional layers are followed by pooling layers to reduce the dimensionality. We use two dropout layers in between the fully connected layers for better regularization. The final prediction is made by a sigmoid activation function and as loss function, we employ standard cross-entropy loss.

For evaluation, we split the labeled data into a training set of ∼28.7 million or 80*%* and a test set with the remaining reviews. The split was performed on a user level such that reviews from one user are all contained either in the training or in the test set. As a result, our classification model is able to correctly classify 75.2*%* of all reviews. For each user, we aggregate in a subsequent step the predicted gender label of all her (his) authored reviews (recall that a gender label is assigned to each review independently) and then assign her (him) a gender behavior score through majority voting. Once a user is labelled, we assign each review the gender of its corresponding author (user). This enhances the gender prediction accuracy to 82.2*%*.

#### Comparison With Human Gender Perception of Review Text

In this work, we assume that in general, humans do not directly identify a reviewer’s gender from her/his review texts, even though advanced machine learning algorithms are capable of nontrivial accuracy. To check this assumption, we randomly sampled 300 (132 performing men and 168 performing women) reviews with disclosed gender from our dataset and asked three volunteers to infer the gender of the author of each review based on its text only, i.e., no user names or additional product info/images were given. Volunteers were provided four options for their responses – “definitely male,” “definitely female,” “probably male” and “probably female.” The final inference is obtained by majority voting.

We present the result of the experiment in Table 2. For 55 cases (which accounts for 18.3% of the total set), agreement could not be reached among the participants regarding the gender of the author. In only 18 cases (accounting for 6%), the participants could judge the author to be “definitely male.” However, 17 of them were indeed signaling men from their user name. Gender was judged to be “probably male” in 75 cases (25%) of which 64 were performing men and 11 were performing women. For 35 (11.7%) cases, the participants assigned the gender of the author to be women and were correct in all cases. Finally 117 (39%) cases were inferred as “probably female” of which 88 were indeed women while 29 were men. Moreover, all these cases were predicted correctly with high probability by our model. This indicates that machines are better at predicting gender from text than humans.

**TABLE 2:** Results from the survey experiment indicate that humans are indeed not very good at predicting gender from text.

	No majority	Definitely male	Probably male	Definitely female	Probably female	Total
Male	22 (16.7%)	17 (12.9%)	64 (48.5%)	0 (0.0%)	29 (21.9%)	132
Female	33 (19.6%)	1 (0.6%)	11 (6.5%)	35 (20.9%)	88 (52.4%)	168
Total	55 (18.3%)	18 (6%)	75 (25%)	35 (11.7%)	117 (39%)	300

#### Categorization of Reviewers

**FIGURE 3:**
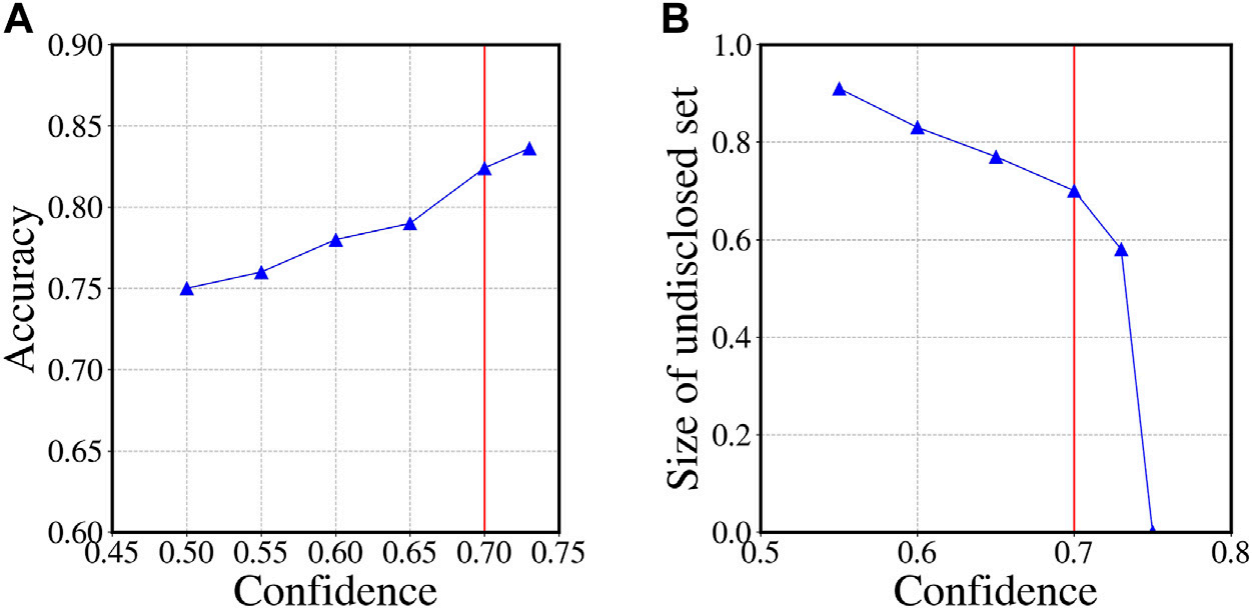
Determining the confidence threshold. **(A)** On labeled test set we plot accuracy versus model confidence. We observe that for a confidence threshold of at least 0.7, we achieve an accuracy of 0.82 and it reduces with lower thresholds. **(B)** For different confidence thresholds we measure the size of the undisclosed set (in fraction). We observe that with a confidence threshold of at least 0.7, the undisclosed set is ∼ 70% of the original set and this reduces drastically as we increase the threshold. Guided by the above result, to strike a balance between accuracy and sufficient size of the undisclosed set, we decide on a confidence threshold of 0.7.

We now leverage the trained model to infer the performed gender of the authors of the reviews in the undisclosed set (i.e., for which the user name does not send a clear gender signal). Note that we only consider the reviews where our model is able to infer gender with probability of at least 0.7 (reduces the undisclosed set to ∼ 29 million). Furthermore, for a given user we aggregate gender information across all her(his) reviews and assign gender through majority voting. The threshold value of 0.7 is guided by the observation that accuracy on the labeled test set starts to decrease with lower values. While a higher value reduces the undisclosed set. This is further illustrated in Figure 3.

The whole set of reviews now can be divided into four categories -

• Signaling (likely) man: Reviews authored by users, for which we can infer that they are likely men from their user name.
• Signaling (likely) woman: Reviews authored by users, for which we can infer that they are likely women from their user name.
• Performing (likely) man: Reviews authored by users who do not signal gender with their user name and for which the text-based classifier identifies them as men with probability of at least 0.7.
• Performing (likely) woman: Reviews authored by users who do not signal gender with their user name and for which the text-based classifier identifies them as men with probability of at least 0.7.

For the sake of readability, we drop “likely” labels from the four categories.

### 2.4 Gender Differences in Perceived Helpfulness

**FIGURE 4:**
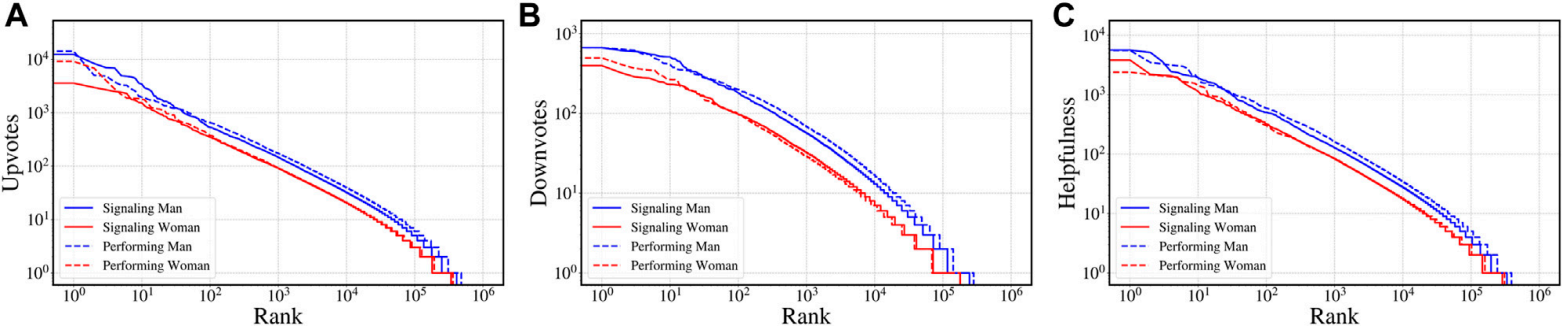
Comparing perceived helpfulness. We sample 1 million reviews from each of the four categories and rank them based on upvotes, downvotes and helpfulness score (#upvotes - #downvotes). We plot the rank and the corresponding values of **(A)** upvotes, **(B)** downvotes and **(C)** helpfulness scores for the sampled reviews. On average reviews authored by signaling men tend to receive more upvotes as well as more downvotes. They are also perceived as more helpful on average.

We now consider the differences in perceived helpfulness between reviews authored by likely men and women as well as differences between users signaling gender versus those performing gender without signaling. To this end, we first sample 1 million reviews from each of the four sets (*signaling men*, *signaling women*, *performing men*, and *performing women*) and then rank them based on upvotes, downvotes and helpfulness score (#upvotes - #downvotes). In Figures 4A–C we plot the rank and the corresponding value for the metrics upvotes, downvotes and helpfulness respectively. We observe that reviews authored by signaling men tend to receive higher upvotes as well as downvotes irrespective of whether gender information is available. Similar observations are made for helpfulness score as well.

## 3 Methods

To adjust for potential confounders in the relationship between gender signaling and perceived helpfulness, we employ matching experiments which we describe next.

### Treatment Groups

Each review in the dataset is classified into one of the four groups - *signaling men*, *signaling women*, *performing men*, and *performing women*.

### Matching Reviews

Ideally, to eliminate potential confounders we would like to compare the perceived helpfulness of a review when authored by one individual of one group (e.g., signaling man) with a review with the same properties when authored by an individual from the other group (e.g., signaling woman). Since a review can only be authored by an individual belonging to exactly one group and it is also unlikely for two reviews to be exactly same, we manually identify a set of potential confounder variables (i.e., variable whose presence affects the outcome of the variable being studied) and control for them. For that purpose, we leverage Mahalanobis matching (Rubin, 1980) on a set of confounders (factors that directly influence the perceived helpfulness of a review) for each review to obtain similar review pairs.

When comparing groups *S*_1_ and *S*_2_, we randomly select a review authored by an individual in *S*_1_ and obtain the most similar review from *S*_2_. Typically, the similarity is measured in terms of Mahalanobis distance, *see* (Mahalanobis, 1936), on the following confounders - *1*) time when the review was published, *2*) length (in terms of number of words), *3*) readability, *4*) sentiment and *5*) overall rating of the product. While *1*) ensures that both reviews on the matched reviews had approximately equal time of exposure *2*), *3*) and *4*) ensure they are of similar quality. We further ensure that the reviews were written on same product category *6*). Note that we use Mahalanobis distance matching as it allows for variable standardization by including sample covariance matrix in distance calculation. We favored Mahalanobis distance matching instead of propensity score matching due to recent findings highlighted in (King and Nielsen, 2019). In particular, the authors demonstrate that propensity score matching can increase imbalance, model dependence, and bias.

### Paired-Treatment Groups

We consider four paired treatment groups, comparing how helpfulness is perceived when:

1. PM-PW a review is authored by a performing man vis-a-vis when authored by a performing woman and user names do not signal gender.
2. SW-SM a review when authored by a signaling woman vis-a-vis when authored by a signaling man, both inferred from user name.
3. SW-PW a review authored by a signaling woman vis-a-vis when authored by a performing woman that does not signal gender.
4. SM-PM a review authored by a signaling man vis-a-vis when authored by a performing man that does not signal gender.

The first case compares the effects of performed gendered behavior on differences in outcomes. The second compares the effects of signaled gender on differences in outcomes. The remaining two cases measure the advantage (resp. penalty) gained (resp. paid) for signaling gender information.

### Balancing Paired-Treatment Groups

First, we randomly sample *N* = 10, 000 instances from the union of all the treatment groups. We use sampling since the following matching procedure is computationally unfeasible for the complete dataset. Moreover, the number of reviews in each category is still large, and hence results on a random sample are still representative for the overall population.

Note that by doing so, the initial random sample is not balanced between the four groups but reflects the overall distribution of the data. Then, we identify for each review the most similar review from each of the other groups across the complete set of reviews, i.e., also outside of the initial sample. So for a group *S*_1_ (e.g., signaling man), we obtain matching reviews from others groups *S*_2_ and *S*_3_ (e.g., signaling women and performing women). We obtain matched review sets for the four paired treatment groups.

### Comparing Outcomes

Note that each pair of reviews obtained for a paired-treatment groups (obtained in previous step) are analogous to each other in terms of review quality and times of exposure and should ideally elicit similar perception among users. For a set of *N* matched reviews for a given paired-treatment group {*S*_1_ × *S*_2_}, we calculate the mean helpfulness score (|upvotes| - |downvotes|) for the reviews in *S*_1_ ( $h_{S_{1}}$ ) and *S*_2_ ( $h_{S_{2}}$ ). The advantage of one group over the other is then denoted as $\frac{\left|h_{S_{2}}-h_{S_{1}}\right|}{\min\left(h_{S_{2}},h_{S_{1}}\right)}\times 100$ . If the gender of the author does not influence how a review is perceived, the advantage should be negligible. To investigate the robustness of the results, we perform bootstrap sampling on the sampled pairs. Typically, for each paired-treatment group, once we have sampled *N* pairs, we perform a sampling with replacement from this *N* sampled pairs. This leads to a resampled set of the *N* pairs. The procedure is then repeated *N* times to obtain *N* bootstrap samples each of size *N*. We report the mean advantage and the standard error calculated over these bootstrap samples.

## 4 Results

As mentioned previously, we consider four paired treatment groups. The experiments are carried out across 15 different categories (selected based on the number of reviews as well as diversity) - *1*) Books, *2*) Electronics, *3*) CDs, *4*) Clothing, *5*) Home, *6*) Kindle, *7*) Sports, *8*) Cellphone, *9*) Toys, *10*) Games, *11*) Literature, *12*) Beauty, *13*) Health, *14*) Movies and *15*) Computers. We now look into each paired treatment group in detail.

**FIGURE 5:**
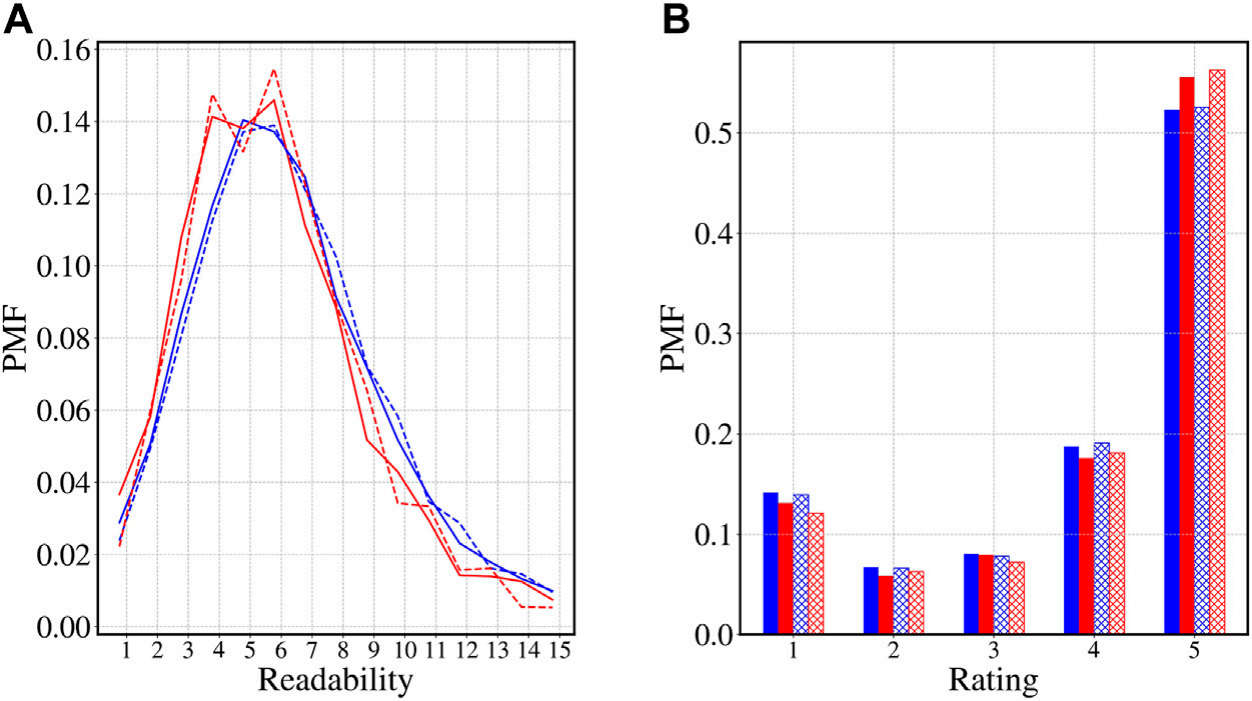
Distribution of the matching confounders. We present the distributions of two exemplary potential confounders **(A)** Readability score and **(B)** overall rating for the reviews in the matched set across the four groups for the category Books. Note that for the two confounder (results are similar for others) the distribution across the four groups closely resemble each other. This shows that the reviews in the matched set are indeed similar and hence inference drawn on these matched sets are consistent.

To start with, we look into the distribution of confounders in the matched sets across the four groups. In Figure 5, we plot the distribution of readability and ratings of the reviews written on Books across the four groups. We observe, the distributions closely resemble each other across all four groups. The results are similar for other confounders as well. This demonstrates the reviews in the matched set are indeed similar and the inferences drawn from the matching experiments are consistent.

**FIGURE 6:**
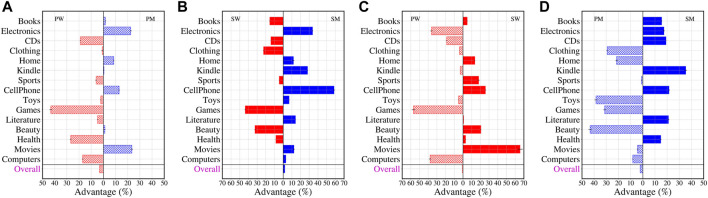
The effects of gender signaling and performance. We show the advantage gained by one group over the other across different categories when comparing the paired-treatment groups - **(A)** performing women and performing men (PW-PM), **(B)** signaling women, signaling men (SW-SM), **(C)** performing women, signaling women (PW-SW) and **(D)** performing men, signaling men (PM-SM). Notably reviewers with unclear gender signals in their user name but performing as women receive higher scores than signaling women in categories such as Electronics, Games or Computers while the same is true for reviewers performing as men with unclear gender signals in categories like Clothing, Beauty or Toys. However, *overall*, there seems to be no effect of gender signaling on the perceived helpfulness of a review in general. Note that in each case the advantage reported is calculated as mean over bootstrap samples. We also report the standard error of the means which are very low for all the cases across four paired-treatment groups.

In Figures 6A–D, we plot the advantage (mean calculated over the bootstrap samples) of one group over the other. We also report the standard error for the average advantages in the same figures. The errors are low in almost all cases. Overall, across all categories we do not find any critical advantage of one group over the other, which means gender signaling does not have a compelling effect on the perceived helpfulness of a review. However, we do observe within-category effects.

### Performing Women - Performing Men (PW-PM)

The comparison of the groups *performing women* and *performing men* allows to analyze if gendered behavior in the absence of explicit signals can be associated with helpfulness perception. We plot the advantage of one group over the other in Figure 6A. Performing men reviewers have advantage over their women counterparts when writing reviews for products on Movies (23.8), Electronics (20.7), Cellphone (14.8) and Home (9.81) while the opposite is observed in case of Games (44.2), Health (27.6) CDs (18.8) and Computers (17.4). For the other categories, the advantage of one group over the other is marginal if any. Overall, across all categories, there is no significant advantage for one group over the other.

### Signaling Women - Signaling Men (SW-SM)

Studying differences between the groups *signaling men* and *signaling women* show differences in helpfulness scores between genders if the gender is signaled *via* user names. Although there does not seem to be any advantage overall for any particular group, we do observe significant advantages across individual categories (refer to Figure 6B). Likely women get higher helpfulness scores in Game (44.9), Beauty (32.1) and Clothing (22.5). Signaling men gain advantages over signaling women in categories Cellphone (60.5), Electronics (35.6), Kindle (28.5) and Movies (12.1). For other categories the advantages are marginal.

### Performing Women - Signaling Women (PW-SW)

Comparing the groups *performing women* and *signaling women* enables the effect of signaling the gender for women under the assumption of a similar writing style (gender performance). We now consider the paired treatment group PW-SW which allows us to probe into whether signaling women do better than performing women reviewers. In Figure 6C we plot the advantage gained by one group over the other across different categories. We note that performing women reviewers have better outcomes than signaling women in categories like Electronics (38.5), Games (56.8) and Computers (38.8). For categories like Movies (62.2), Cellphone (25.2) and Beauty (19.8) the opposite effect is observed. For other categories the advantage is marginal if any. However, there is no advantage for any group on average overall.

### Performing Men - Signaling Men (PM-SM)

Analogously, comparing the groups *performing men* and *signaling men* allows for investigating the effect of signaling the gender for men under the assumption of a similar writing style (gender performance). Finally, we investigate differences in helpfulness scores between signaling men and performing men reviews. We observe (refer to Figure 6D) that signaling men do better than performing men reviewers in categories like Kindle (33.6), Cellphone (21.6), CDs (17.5) and Electronics (16.4). In categories like Beauty (42.4), Toys (35.3), Clothing (29.1) and Games (31.2) the opposite is true. Again, we observe only negligible advantage overall on average.

## 5 Discussion

In this section we present the implications as well as few limitations of our study.

### 5.1 Implications of Results

For categories like Electronics, we observe that the signaling women get less positive feedback than similar signaling men. They also get less positive feedback than performing women. This suggests a disclosure penalty, i.e. that other users consider the signal encoded user names when judging review helpfulness. Similarly, for categories such as Beauty, signaling men are at disadvantage. One potential mechanism for this effect is that users judging reviews apply gender stereotypes–for example, that men are more knowledgeable about electronics while women are better informed about beauty products–to rate reviewers when they can infer gender from names. As these ratings influence the ranking and visibility of reviews to shoppers, this can amplify stereotypes.

**FIGURE 7:**
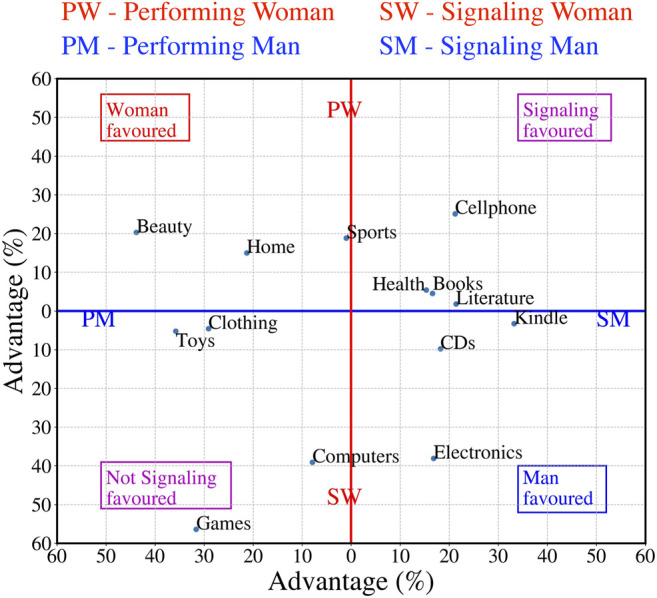
Summary of the context-specific gender effects. We combine and summarize the results of Figures 6C,D to classify each category into - *1*) signaling man favoured, *2*) signaling woman favoured, *3*) signaling favoured and *4*) performance favoured. The *x*-axis from left to right denote performing to signaling men (PM - SM) while *y*-axis from top to bottom denotes performing to signaling women (PW - SW). Each point in the figure denotes a category and its position is determined by its corresponding value in Figures 6C,D. Notably, categories like Electronics, CDs or Kindle seem to favour signaling men. Similarly Beauty or Home seem to favor signaling women. Moreover, categories such as Cellphone, Health or Books favour gender signals from user names while Games, Computers and Toy favour non-signaling.

There is no global advantage or disadvantage for those users whose avatars do not signal a gender. However, such effects are observed within individual categories. We classify each category into one of four groups - *1*) signaling men favoured, *2*) signaling women favoured, *3*) gender-signalled favoured, and *4*) non-disclosure favoured based on their advantage score for (un)disclosing gender information (refer to Figures 6C,D). For example in Electronics category signaling men hold an advantage of 16.4 over performing men while performing women secure an advantage of 38.4 over their signaling women counterparts. This places the Electronics in the quadrant man favoured with coordinates (16.4, 38.5). We make a few observations:

• Categories like Beauty and Home seem to favour women as gender signaling *via* names increases helpfulness compared with performed gender while performing men do better than their signaling counterparts. The exact opposite holds for categories such as Electronics, CDs and kindle (refer to Figure 7).
• Categories like Cellphone, Health and Books seem to favour users signaling gender *via* their names. Similarly categories such as Computers, Games and Clothing seem to favour gendered performance without signals from user names (refer to Figure 7).

### 5.2 Limitations

#### User Names and Real Gender

We generally consider that the user names signal the true gender of the user. This assumption has been adopted previously in multiple studies and has shown practical relevance, despite various shortcomings (Keyes, 2018). A second simplifying assumption we make is that gender is binary. As we are measuring the social feedback received by reviewers, the salient gender feature is how a user’s gender is perceived, rather than how the user identifies. A greater matter of concern is the known western bias of name-based gender inference tools including gender-guesser (Qiu et al., 2019). As our data comes from a platform based in North America and gender-guesser has repeatedly been evaluated with good performance for western names (Karimi et al., 2016; Santamaría and Mihaljević, 2018), we argue that this limitation is acceptable, but we certainly acknowledge that extension of our analyses to other regions will require careful modification of the gender inference approach.

#### Disclosure of Gender

One important point to consider is whether usernames are considered at all while assessing the helpfulness of a review. Past work using surveys and eye-tracking software indicate that users do notice and reflect on social signals when evaluating online content (Marlow et al., 2013; Ford et al., 2019). Although our results suggest there is indeed a dependency between gender signaling and perceived helpfulness, further analysis is required in this regard. In fact, our results may suggest that some users face incentives to hide or conceal their gender (Vasilescu et al., 2012).

#### Inferred Gender Behavior Through Machine Learning

For the undisclosed set we utilize our trained machine learning model to infer gendered behavior from the text of the reviews. Although our model seems to perform well (accuracy of 82.2*%*) on the ground truth (disclosed set), we cannot assess its performance on the undisclosed set. Likely there is a natural limit to the extent to which behavior conforms to gender identity. This limit is probably highly dependent on socio-cultural norms and so is changing all the time. Implicit in our approach is the assumption that gender signals from user names and gender performance measured from content align or overlap. Future work is needed to better understand the complex interaction between presentation and performance of gender and its effects on online feedback.

#### The Mechanisms Behind Observed Differences

Our experiments cannot reveal why disclosing or performing gender relates to different outcomes in different characters. While our findings do suggest that sometimes signaling gender does relate with better outcomes, it is unclear if this is because of audience demographics and preferences or bias. More work is needed to understand the process by which individuals rate reviews.

#### Experimental Limitations

In this work we have used Mahalanobis Distance Matching as the preferred matching method. However, to test the robustness of the method one needs to look into *additional matching methods* like coarse exact matching or caliper-based approaches. Moreover, we consider a set of six confounders of which readability, sentiment and length are selected to determine the quality of the review. However, more specific linguistic and psychological (presence of “insightful,” “causation” or “inclusive” words) dimensions could be used as *additional confounders* as well.

## 6 Related Work

The Web provides us a giant platform for observing human behavior and allows us to answer various socially relevant questions. With increasing availability of large amount social media data, a significant amount of research efforts have been directed towards understanding human behavior from this data (Ruths and Pfeffer, 2014). Consequently, research efforts have been able to identify compelling evidence towards the presence of gender inequality in different aspects of social media.

### Gender Inequality

In (Wagner et al., 2015), the authors explore the gender inequalities on Wikipedia and observe that women are portrayed in a starkly different way than men. Gender and racial biases are also observed in online freelance market places (Hannák et al., 2017). In (Nilizadeh et al., 2016), the authors find that women are underrepresented among the top users on Twitter. A similar effect was documented in the content of online newspapers (Jia et al., 2016). These online gender biases can have significant economic consequences (Foong et al., 2018). Sometimes the design of platforms amplify gender differences, suggesting potential points of intervention. On Stack Overflow, a Q&A platform for programmers, men and women have significant differences in behavior, and platform design choices translate this difference in behavior to a gap in outcomes (May et al., 2019).

### Gender Perception

The gaps and disparities described above suggest that users in online communities make assumptions and stereotypes about contributions using socio-demographic features such as age, gender, and ethnicity (Willis and Todorov, 2006). In (Davison and Burke, 2000) the authors note that men and women job applicants receive lower ratings for jobs stereotypically held by members of the other gender (e.g. nurses and carpenters). Men are also often rated more competent than women purely based on cues of gender and not content (Fiske et al., 2018). An analysis of Github, a platform for collaborative programming, using a dataset with self-reported gender identification suggests that contributions of women are accepted more often when their gender is hidden (Terrell et al., 2017). (Ekstrand and Kluver, 2021) explore gender perception in book rating and recommendation albeit from recommendation algorithm perspective while (Choi and Horvát, 2019) investigate gender differences in a prominent home sharing platform Airbnb.

### Gender Performance

Our paper contributes to a growing area of research that describes how gendered performance impacts success and reception. Past work by Otterbacher describes differences in the writing style of IMDB film reviews between men and women (Otterbacher, 2010). Otterbacher finds that “feminine” reviews are typically ranked as less helpful. More recent work extends the measurement of gendered behavior to visual content creation (Wachs et al., 2017), music performance (Wang and Horvát, 2019), and software engineering (Vedres and Vasarhelyi, 2019). A consistent finding across these domains is that individuals are less successful when they create content with a more feminine style, regardless of their signaled gender (Brooke, 2019).

### Gender and Reviews

(Bae and Lee, 2011) examine whether there are gender differences in responding to online consumer reviews. In particular, they observe that women tend to be more influenced by negative reviews than positive reviews while making purchase decisions. Through web-based experiments (Craciun and Moore, 2019) observe that when reputation cues are present, emotional content in reviews lowers the credibility of men reviewers as well as the helpfulness scores on their reviews while women reviewers are not affected. (Zhang et al., 2014) observe that the influence of emotional trust on purchase intention is significantly stronger if the reviews are inconsistent. In fact, the effect is stronger for women consumers. Gendered effects on reviews have also been studied across other platforms - video games (Ivory, 2006), hotel (Kim et al., 2011) and travel (Assaker, 2020).

Note that most of existing work are aimed at understanding gender differences across different online platforms. In contrast, we are more interested in understanding whether an advantage in terms of perceived helpfulness, is to be gained by (not) signaling gender through gendered user names. Our work also contributes to an ongoing discussion about whether or not users have incentive to obscure their gender online. As mentioned above, Terrell et al. find evidence that women may be more successful when they hide their genders on Github Terrell et al. (2017). What is clear is that anonymity has an important influence on how individuals perform and are evaluated online Paskuda and Lewkowicz (2015, 2017); gender is just one dimension of this phenomenon.

## 7 Conclusion

In this work, we have quantified the effect of gender signaling on perceived helpfulness of reviews in a large online shopping platform.

For our analysis, we employed a dictionary based name-to-gender tool to infer gender signals, character-level Convolutional Neural Networks to characterize gender performance, and Mahalanobis matching to measure relationships between these gender features and success. We observed basically no general effect for either gender signaling or performance. Rather, we saw substantial category-specific effects: reviews authored by signaling women are perceived as more helpful in categories like Toys, Movies and Beauty while signaling men receive more kudos for their contributions to categories including Electronics, Kindle, and Computers. In the second dimension of our analysis, we found that in categories like Electronics or Cellphones gender anonymous reviewers performing as women receive better feedback than signaling women. Similar effects are observed for reviewers performing as men compared with signaling men in categories such as Books or Kindle.

In the future, it will be interesting to extend our idea to other web platforms and thereby investigate whether gender disclosure and signaling has effects on perceived helpfulness in other domains. The task of inferring gender or gendered behavior from text is a fascinating problem in its own right and demands further inquiry. Moreover, we suggest that future work ought to explore the signaling of race and its influence on how content is received online. Finally, analyzing longitudinal trends in the observed effects could be an interesting direction for future research.

## 8 Contribution to the Field Statement

Our work demonstrates that the presence of context-sensitive gender related effects (e.g., reviews in product categories such as “Electronics” or “Computers”) are perceived as less helpful when authors signal that they are likely woman but are received as more helpful in categories such as “Beauty” or “Clothing”) which uncover through analyzing ∼80M reviews on “amazon.com.” We believe that this significantly extends previous findings in the field. Our results promote increased awareness of gender-specific effects in the perception of online reviews and suggest implications for online platforms (e.g., regarding the ranking of reviews or display of user names if they potentially imply gender) and their users (e.g., awareness of such effects when choosing a user name). The ensemble of tools and methods that combines state-of-the-art machine learning (e.g., deep learning-based text classification) and classical statistical techniques (e.g., matching algorithms) will also enable other researchers to easily measure gender specific effects across a variety of online social media platforms. Our work illuminates a way towards a deeper understanding of the subtle effects of gender signals while modeling user behavior on the Web.

## Data Availability

The dataset was gathered from https://jmcauley.ucsd.edu/data/amazon/. Publicly available datasets were analyzed in this study. This data can be found here: https://jmcauley.ucsd.edu/data/amazon/.

## Footnotes

1
https://www.amazon.com

2
https://pypi.org/project/gender-guesser/

3
http://jmcauley.ucsd.edu/data/amazon/, “aggressively deduplicated data” version

4
https://pypi.org/project/gender-guesser/
